# Research and Remedies

**Published:** 1914-03-14

**Authors:** 


### RESEARCH AND REMEDIES.

### A Unique Surgical Feat.

A splendid feat of bold surgery, followed by the happiest results, is reported by Dr. Bauer in the *Zentralblatt für Chirurgie*. A patient in the Malmö Hospital (Sweden), suffering from mitral stenosis and regurgitation, was seized by sudden pain and paralysis in both legs, which at once became cold and cyanosed. A diagnosis of embolism of the aorta was made, and it was deduced that the embolus must have lodged near the bifurcation of the aorta, because there were no symptoms referable to the bowel or kidneys. No pulse could be felt anywhere in either leg. Accordingly, preparations for operation were made, and three hours after the first onset of symptoms a median laparotomy was performed. It was found that the abdominal aorta was pervious and pulsating down to about an inch from its division into the two common iliac trunks, but not any further. An assistant compressed the artery against the vertebral column, and Bauer opened it by an incision three-quarters of an inch in length. A large embolus was extracted and the aorta sewn up again by Carrel's method. Both iliac arteries at once pulsated, and the patient made perfect recovery. But for the timely surgical interference it is thought he must have developed gangrene of both legs, to say nothing of the bladder. The embolus was more than an inch long, and possessed two branches, one from each iliac artery. It possessed a nucleus of old formation, around which was a mass of fresh blood clot. It is stated that this is the first case of this kind operated on successfully.

### Malta Fever in London?

Major J. C. Kennedy has put on record in the *Journal of the Royal Army Medical Corps* certain facts which suggest this query. Without wishing to be an alarmist, he nevertheless does commit himself to the fact that some London milk agglutinates the Malta fever bacillus. This, of course, places these cows and their milk immediately under the gravest suspicion, and it is to be hoped the matter will be followed up. Major Kennedy, unfortunately, has gone abroad on service, so to someone else the duty must be delegated. It will be remembered that the very serious epidemics of Malta fever in our Mediterranean garrisons were ultimately traced to the milk of infected goats, since when the epidemics have practically ceased from troubling the Army and Navy authorities. As yet the bacillus has not been isolated from the cows whose milk and serum reacts in such a way as to suggest that they are infected. But the account of the bacteriological work makes it quite clear that these cows must be looked upon with grave suspicion.

